# Soft tissue regeneration around immediate implant placement utilizing a platelet-rich fibrin membrane and without tightly flap closure: Two case reports

**DOI:** 10.1097/MD.0000000000022507

**Published:** 2020-10-02

**Authors:** Aimin Cui, Jing Zhou, Mahmoud Mudalal, Yao Wang, Jia Wang, Ming Gong, Yanmin Zhou

**Affiliations:** a Department of Oral Implantology, School and Hospital of Stomatology, Jilin University, Changchun; b School of Stomatology, Hospital of Stomatology, Tianjin Medical University, Tianjin, China.

**Keywords:** immediate implant replacement, platelet-rich fibrin, semi-open flap, soft tissue regeneration

## Abstract

**Rationale:**

In this report, a combination of platelet-rich fibrin (PRF) membrane and semi-open flap technique was used to improve soft tissue regeneration in immediate implant placement in the molar region. PRF, an autologous fibrin matrix, has been widely used for soft tissue wound healing and regeneration. Semi-open flap technique is beneficial to eliminating exudates and relieving the swelling after surgery.

**Patient concerns:**

Case 1 was a 45-year-old female with a residual crown in the posterior maxillary region that desired a dental implant operation. Case 2 was a 24-year-old male with retained deciduous tooth that requested a restoration of his congenital absent tooth.

**Diagnoses:**

In case 1, the tooth 16 was diagnosed with a residual crown, while in case 2, a deciduous tooth 75 was a retained deciduous tooth and 35 was congenital absent.

**Interventions:**

In both cases, immediate implant placement was installed and PRF membranes were made to improve soft tissue augmentation with semi-open flap technique. In case 1, the mixture of an organic bovine bone and blood was filled in the gap between the implant and the socket wall. Subsequently, 2 PRF membranes covered the open wound with semi-open flap. Similarly, in case 2, another 2 PRF membranes were used to improve the soft tissue regeneration, with the same semi-open flap technique as mentioned above.

**Outcomes:**

In both cases, successfully soft tissue regeneration was obviously observed without postoperative infection.

**Lessons:**

Utilizing the PRF membrane combined with semi-open flap technique can achieve excellent soft tissue augmentation around immediate implant placement in the molar regions.

## 1 Introduction

Currently, immediate implant placement is acknowledged as a common technique with good clinical effects. The overall implant survival rate was 96.0% in a retrospective review of 891 cases with 1925 immediate implant placement from 1988 to 2004.^[1]^ Compared with traditional implant treatment, immediate implant placement has many advantages such as decreasing trauma, shortening the treatment period and reserving the alveolar bone. However, after extracting the molars, the absence of soft tissue can easily be found.

Platelet-rich fibrin (PRF), an immune and platelet concentrate, has been proved to have a good effect on soft tissue healing and regeneration. PRF has a three-dimensional structure of the fibrin matrix which holds and releases cytokines and growth factors including platelet-derived growth factor (PDGF), transforming growth factor (TGF), vascular endothelial growth factor (VEGF), and insulin-like growth factor (IGF).^[2]^ These factors are essential to regulate integrin expression, proliferation and differentiation of mesenchymal stem cell and their migration inside the wound.^[2]^

Most previous clinical studies confirmed that tight flap closure around implants appears to be a significant procedure in surgery.^[3]^ This may promote reattachment and block bacterial invasion. However, recently, clinical experiments showed that the absence of primary flap closure did not affect the percentage of vital bone formation or residual graft^[4]^ and may have less postoperative discomfort and swelling. Up to now, we didn’t receive any specific researches to decide which suturing technique is better to wound healing.

This report showed 2 typical clinical cases in an immediate implant placement in the molar region. PRF membranes were made to regenerate the soft tissue around the implants. On this basis, the traditional implant surgery was slightly modified. Instead of tight suture, semi-open flap (loose stiches) was performed and eventually achieved ideal regeneration effect.

## 2 Case report

For both cases, informed consent agreements were obtained from the patients for publication of case reports and accompanying images before surgery. Furthermore, parameters assessed to check the inflammation and expected outcome were carried out at the suture removal and second stage surgery visits. Surgical sites were examined carefully at the suture removal visit, and the patient was asked about any other postoperative discomfort or swelling. During the second stage surgery peri-apical x-rays were established to check the healing and osteointegration status of the surgical site.

### 2.1 Case 1

**Figure 1:**
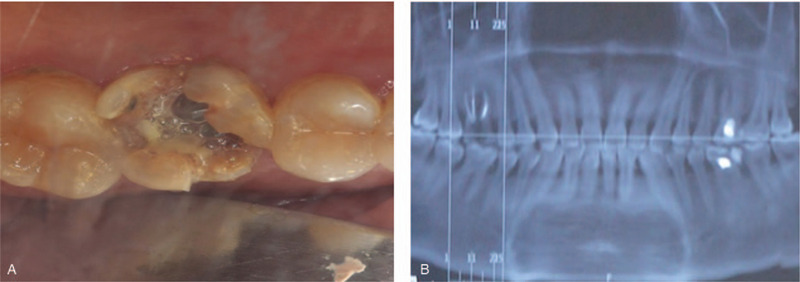
Preoperative and intraoperative photographs of case 1. (A) Clinical view of the tooth 16; (B) Radiographic aspect of the tooth.

A 45-year-old female patient came to the Oral Implantology Department of School and Hospital of Stomatology at Jilin University for implant placement. Upon clinical examination, the maxillary right first molar was irremediable with a residual crown. The radiographic examination revealed that root filling materials were found in 16 root canals (the tooth was numbered according to the FDI World Dental Federation notation) (Fig. 1A and B). Based on the condition above, a treatment procedure with immediate implant placement without flap release was planned, simultaneous bone, soft tissue augmentation, and PRF membrane without primary closure were established.

**Figure 2:**
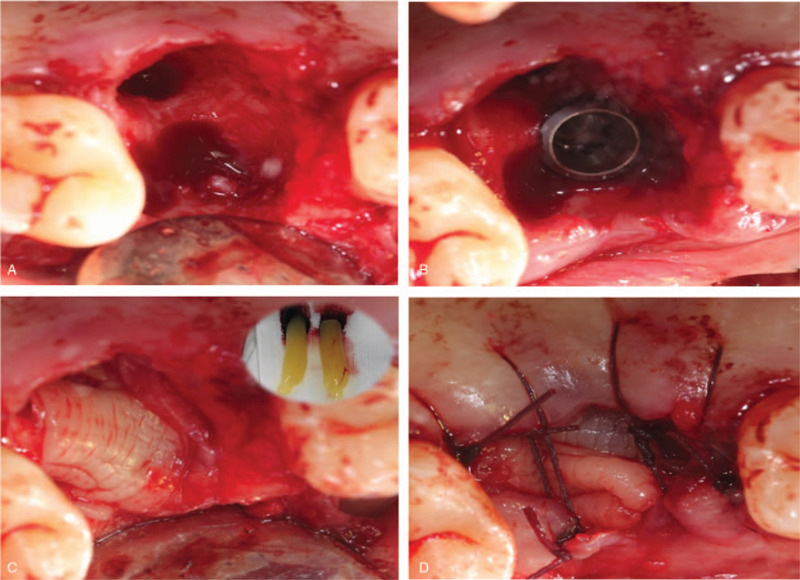
Intraoperative photographs and illustration describing each step of the surgery. (A) The tooth was carefully removed; (B) A bone defect gap around the implant; (C) 2 PRF membranes covered over the bone materials; (D) Semi-open flap was used. PRF = platelet-rich fibrin.

**Figure 3:**
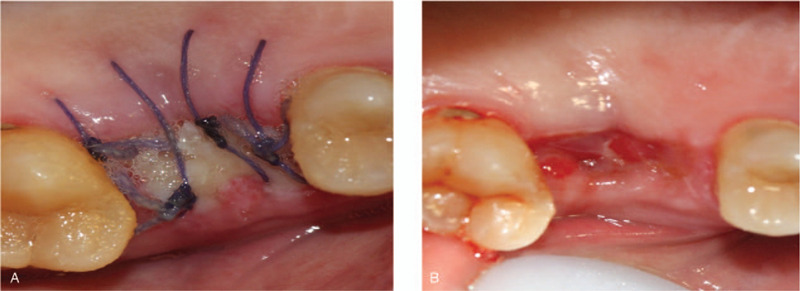
Postoperative photos of case 1. (A) Clinical view of surgery site at 7 days follow-up. (B) Clinical view of surgery site at fourteen days follow-up.

Before surgery, mouth rinsing with 0.12% chlorhexidine solution was performed 3 min/time for three times. After local anesthesia, 16 was extracted with minimal invasion and all granulation tissue was curetted from the tooth socket. Meanwhile, 20 mL of whole blood was collected in 2 separate glass-coated 10 mL plastic tubes without an anticoagulant from the patient. After that, the blood samples were immediately centrifuged at 3000 rpm for 10 minutes for preparation. Implant preparation was conventionally drilled and an implant (Astra Tech, Sweden, Φ4.5 mm × 10 mm) was placed in the alveolar socket with a torque of 35N/cm, following with a cover screw placement. An obvious bone defect gap was observed around implant (Fig. 2A and B) and the mixture of 0.25 g an organic bovine bone (Bio-Oss, Geistlich, Switzerland) and blood were filled into the defect area and over the implant. Two PRF clots were pressed into membranes and covered over the bone materials. Semi-open flap was used instead of tight 1 (Fig. 2C and D). The patient received post-surgical care including amoxicillin tablets 0.5 g and metronidazole 0.3 g 3 times per day for following 3 days for oral administration. Chlorexidine (0.12%) was used for mouthwash twice per day for 7 days following the instruction. The sutures were taken out 7 days later with no inflammation (Fig. 3A). The patient denied swelling and pain after the surgery. Fourteen days follow-up visit, the wound was well healing and the vascularization of soft tissue was visible (Fig. 3B).

**Figure 4:**
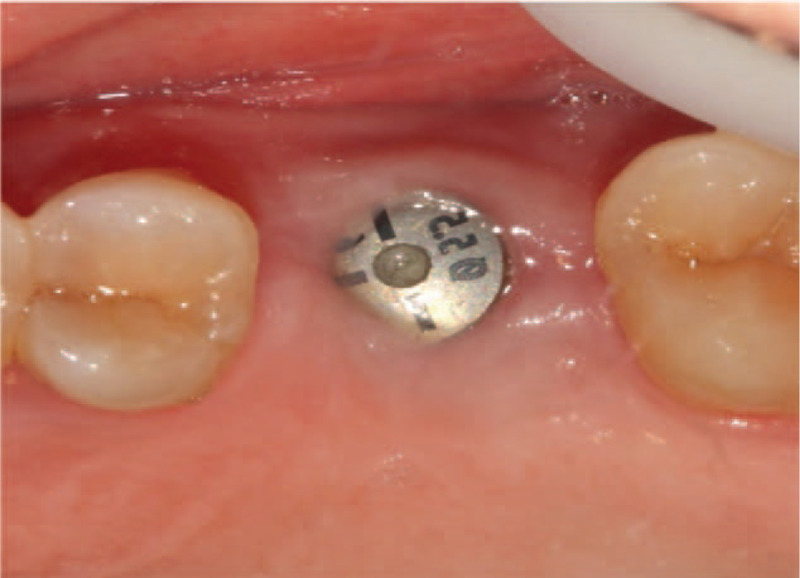
Clinical view of surgery site after second-stage surgery.

**Figure 5:**
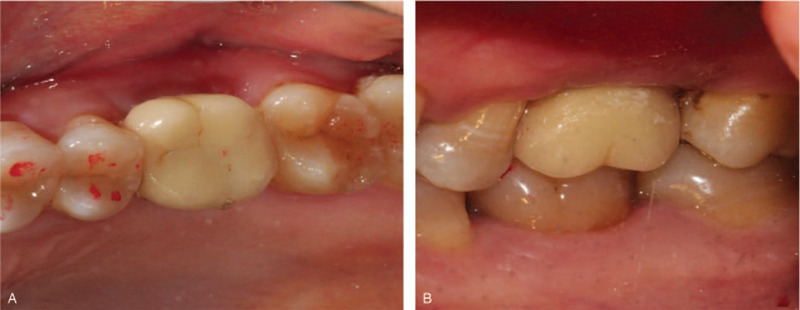
A zirconia ceramic crown was constructed: (A) Occlusal view of the final prosthesis in place; (B) Buccal view of the final prosthesis in place.

Second-stage surgery was performed 6 months after surgery. Intraoral examination revealed soft tissue volume were sufficient compared with pre-surgery. Flap operation and bone removal over the healing abutment were performed. A healing abutment were changed with a tight closure. Finally, the soft tissue healed well and the keratinized gingiva around the implant were more than 2 mm (Fig. 4). Then, a zirconia ceramic crown was constructed (Fig. 5A and B).

### 2.2 Case 2

**Figure 6:**
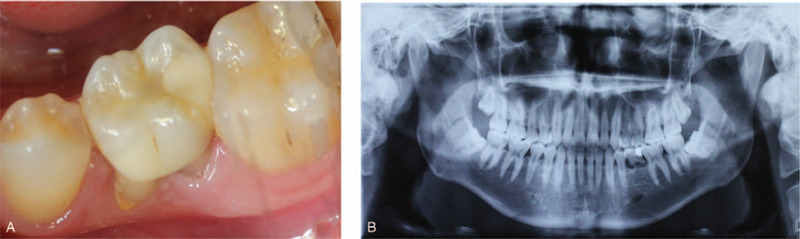
Preoperative and intraoperative photographs of case 2. (A) Clinical view of the tooth 75; (B) radiographic aspect of the tooth.

A 24-year-old male patient came to the Oral Implantology Department of School and Hospital of Stomatology at Jilin University for implant placement. Clinical examination and radiographic examination revealed that deciduous tooth 75 was retained and tooth 35 was congenitally absent (Fig. 6A and B). The patient had good condition without any contraindications to implant surgery. The treatment plan was performed with routine implant placement, soft tissue augmentation with the PRF membrane and without primary closure was planned.

**Figure 7:**
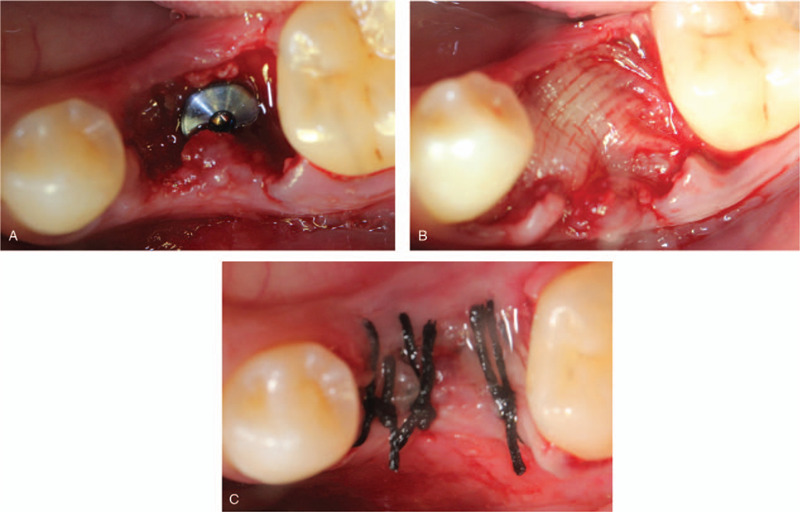
Intraoperative photographs and illustration describing each step of the surgery. (A) The implant was placed; (B) 2 PRF membranes covered over the surgery site; (C) Semi-open flap was used. PRF = platelet-rich fibrin.

**Figure 8:**
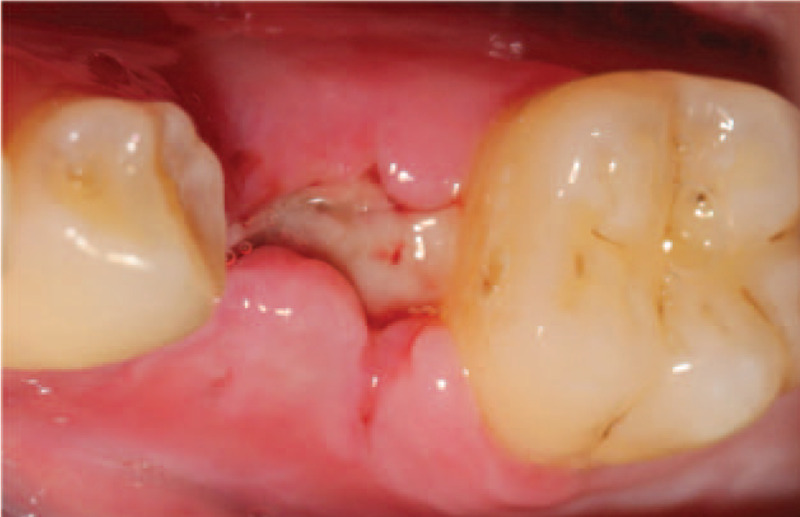
Clinical view of surgery site at 8 days follow-up.

Before surgery, patient rinsed with 0.12% chlorhexidine solution for 3 min/t for 3 times. After local anesthesia, 75 was extracted and meanwhile, PRF was prepared followed the same protocols as case 1. After that, implant bed preparation was drilled step by step and an implant (Nobel Replace, Sweden, Φ5.0 mm × 10 mm) was placed in the alveolar socket with a torque of 35N/cm, thereafter, a healing abutment was placed. Two PRF clots were pressed into membranes and covered over the cut. Similarly, semi-open flap was used (Fig. 7A, B, and C). Post-surgical instructions were performed as the case 1. The sutures were taken out 8 days later with no inflammation records (Fig. 8). The patient denied swelling and pain after the surgery.

**Figure 9:**
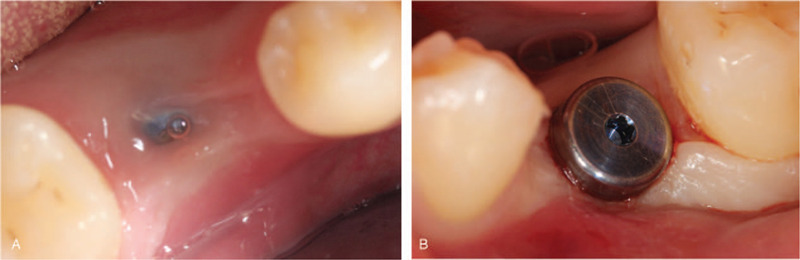
Clinical view of surgery site at three-month follow-up. (A) Clinical view before the second stage surgery; (B) Clinical view after the second stage surgery.

Second-stage surgery was performed 3 months after surgery. Intraoral examination revealed the keratinized gingiva volume were sufficient (Fig. 9A and B). A higher healing abutment were changed without suture.

## 3 Discussion

Due to large extraction sockets in the posterior tooth, soft tissue defects are often found over the implant. Suturing the wound directly can cause flaps with excessive tension which may result in wound dehiscence.^[5]^ This can cause delayed healing, leakage of bone graft materials, and increase possibility of infection. Local soft tissue flaps are a common method for the treatment of soft tissue defect around implants. However, because of thinner gingiva or mucosa, flap perforation may occur during the flap surgery. Free autogenous connective tissue grafts or free autogenous gingival grafts are also common methods for soft tissue contour augmentation.[^6^,^7^,^8^] Since volume of connective tissue is limited by anatomical parameters, soft tissue augmentation effect is inadequate. Furthermore, subsequent complications may develop such as massive bleeding, poor wound healing and infection. PRF, a second-generation platelet concentrate, is regarded as a natural fibrin-based biomaterial with 3 dimensional mesh structure and contains various growing factors.^[9]^ PRF was reported to have characteristics of accelerating tissue cicatrization during the process of effective neovascularization, accelerating wound closing with fast cicatricial tissue remodeling, and having no pain, dryness or purulent complications of the wounds.^[10]^ Recently in the dental field, PRF was reported to have great effect on treatment of gingival recessions,^[11]^ regeneration of periodontal defects,^[12]^ oral mucosal lesions^[13]^ and palatal wound closure.^[14]^ Therefore, the advantages of such membranes are obvious to protect open wounds and accelerate healing.

In the past, most authors agreed primary wound closure was desirable especially in guided bone regeneration procedure,^[15]^ while some authors did not think it to be of great relevance.^[16]^ Waasdorp J et al reported 3 successful clinical cases of dense polytetrafluoroethylene membrane without primary closure for the bone regeneration around immediate implants.^[17]^ They thought that obtaining primary closure through coronally advanced and pedicled flaps may disrupt part of the blood supply, companied with soft tissue structure of the area altered and the surgical complexity increased.^[17]^ Non-resorbable polytetrafluoroethylene is impenetrable to bacteria and epithelial which made it possible for open wound without inflammation. Although PRF is a resorbable membrane, it appears to have a lot of similar characteristics. First, PRF has 3-dimensional mesh architecture which contains denser fibrin. Compared with using collagen barrier membrane alone, PRF shows potential advantages of further regeneration by additional use or replacing the collagen barrier membrane.^[18]^ Second, PRF can release various cytokines which play important roles in regulation of inflammation and post-surgery infection. Hoaglin, et al reported that after placing PRF in mandibular third-molar extraction sites, the incidence of localized osteitis was 1% compared with untreated with PRF patients showing 9.5%.^[19]^ Besides, that PRF can induce the proliferation of dermal fibroblasts, gingival fibroblasts, and keratinocytes, as well as participates in the synthesis of extracellular matrix collagen 1.^[2]^ These properties can prevent bacteria into open wound and promote tissue healing.

Based on mentioned above, we proposed an approach of soft tissue augmentation around molar immediate implant by PRF membrane without primary closure. As we know, the environment of oral cavity is diversity which promotes distinct microbial communities and over 500 microorganism species have been isolated from the cavity.^[20]^ After surgery, since the molar region located very deep, it is hard to keep clean which contributes to the inhabitation of bacteria. On the 1 hand, the conventional tight suture can inhibit bacteria entering the wound. while on the other hand, once the bacteria invade in the wound, the environment of the tight 1 may produce a relative anaerobic environment which promotes the grow of oral anaerobic bacteria. On the contrary, semi-open suture provides an open environment. Moreover, it is conducive to eliminating exudates and relieves the swelling after surgery. In 2 cases, whether bone graft material was added, the wound showed no sign of infection and healed well without swelling when removing the sutures. At 6 months after operation, sufficient volume of keratinized gingiva was obtained and the epithelial cuff healed well. These cases showed successful application of this modified approach and provided a new idea for further clinical use.

## 4 Conclusion

In these 2 cases, we reported an approach of soft tissue augmentation around molar immediate implant using PRF membrane without primary closure. PRF may act as a barrier to prevent bacterial invasion into the open wound. Semi-open flap technique showed us tremendous wound healing with no post-operative infection. With the help of PRF, the effect of keratinized gingival augmentation was considerable. Therefore, this improved approach is feasible in clinical treatment, but more cases should be involved and long-term effects need to be observed.

## Acknowledgment

The authors thank Dr Xiaolin Sun for the fruitful discussion and technique help.

## Author contributions

**Conceptualization:** Yanmin Zhou.

**Data curation:** Jia Wang, Ming Gong.

**Formal analysis:** Mahmoud Mudalal, Yao Wang.

**Project administration:** Jia Wang.

**Supervision:** Yanmin Zhou.

**Writing – original draft:** Aimin Cui, Jing Zhou.

**Writing – review & editing:** Yanmin Zhou.
